# Diagnostic Yield of Opportunistic Upper Gastrointestinal Endoscopy During Elective Colonoscopy in Asymptomatic Patients: A Retrospective Cross-Sectional Study

**DOI:** 10.7759/cureus.118181

**Published:** 2026-10-19

**Authors:** Caroline Assis Aleixo Chaves Freitas, Sandro Rodrigues Chaves, Rafael P Pessoa, Lucas Gallo de Alvarenga Mafra, Júlia Gallo de Alvarenga Mafra, Eduardo Seubert Coelho Vieira, Gabriela Vieira Brito, Giovanna De Giuli, Rodrigo Roda

**Affiliations:** 1 Endoscopy Division, Rede Mater Dei de Saúde, Belo Horizonte, BRA; 2 Gastroenterology Division, Rede Mater Dei de Saúde, Belo Horizonte, BRA; 3 Endoscopy Division, Faculdade Ciências Médicas de Minas Gerais, Belo Horizonte, BRA; 4 Endoscopy Division, Instituto Alfa de Gastroenterologia, Hospital das Clínicas da Universidade Federal de Minas Gerais, Belo Horizonte, BRA

**Keywords:** colonoscopy, diagnostic yield, gastric premalignant lesions, helicobacter pylori, upper gastrointestinal endoscopy

## Abstract

Background

Opportunistic upper gastrointestinal endoscopy (EGD) during elective colonoscopy has been proposed as a strategy for detecting upper gastrointestinal abnormalities in asymptomatic individuals. However, evidence regarding its diagnostic yield and clinical relevance remains limited, particularly in Western populations. This study aimed to evaluate the diagnostic yield of opportunistic EGD performed during elective colonoscopy in asymptomatic patients.

Methods

This retrospective cross-sectional study included asymptomatic patients who underwent opportunistic EGD during elective colonoscopy at a tertiary endoscopy center in Brazil between March 2023 and March 2025. The primary outcome was the proportion of patients with at least one clinically significant finding, including selected premalignant and malignant lesions, *Helicobacter pylori* (*H. pylori*) infection, and other potentially relevant upper gastrointestinal abnormalities. Endoscopic and histopathological findings were retrospectively reviewed. Descriptive statistical analyses were performed, and diagnostic yield was reported with a 95% confidence interval (CI). Changes in clinical management were not assessed.

Results

A total of 421 asymptomatic patients were included, of whom 269 (63.9%) were female; the mean age was 58.4 years. At least one clinically significant finding was identified in 188 patients (44.7%; 95% CI: 39.8%-49.5%). Gastritis was identified in 293 patients (69.6%), and *H. pylori* infection was detected in 102 (24.2%). Premalignant gastric findings included intestinal metaplasia in 45 patients (10.7%), gastric atrophy in 27 (6.4%), and low-grade dysplasia in 2 (0.5%). Gastric carcinoma and mucosa-associated lymphoid tissue (MALT) lymphoma were each diagnosed in one patient (0.2%). Additional findings included gastric polyps in 140 patients (33.3%), esophagitis in 134 (31.8%), bulboduodenitis in 26 (6.2%), and Barrett’s esophagus in 3 (0.7%).

Conclusions

Opportunistic EGD performed during elective colonoscopy demonstrated a substantial diagnostic yield in asymptomatic patients, including the detection of premalignant and malignant upper gastrointestinal lesions. However, the absence of systematic follow-up and a comparison group precludes conclusions regarding actual changes in clinical management or improvements in patient outcomes. Prospective comparative studies incorporating longitudinal follow-up, procedural safety, and economic evaluation are needed to determine the clinical value of this strategy.

## Introduction

Preventive medicine has become one of the main pillars of modern healthcare systems. Given the high prevalence of colorectal cancer worldwide, screening colonoscopy has become a well-established strategy in many countries [1]. With the increasing standardization of this procedure, there has also been growing interest in the simultaneous performance of upper gastrointestinal endoscopy (EGD) during scheduled colonoscopy. This scenario has raised important discussions regarding the potential role of EGD in screening and the appropriateness of its routine use in asymptomatic individuals.

Structural lesions of the upper gastrointestinal tract, including esophagitis, Barrett’s esophagus, gastritis, peptic ulcer disease, duodenitis, polyps, subepithelial lesions, premalignant conditions, and neoplasms, can be detected by EGD. These conditions may be symptomatic or remain clinically silent, potentially presenting later with complications [2]. In addition to identifying premalignant lesions and other upper gastrointestinal abnormalities, EGD enables tissue sampling for histopathological evaluation and detection of *Helicobacter pylori* (*H. pylori*) infection. However, the clinical relevance of these findings varies, and their detection does not necessarily translate into improved patient outcomes.

Gastric cancer remains a major global health concern, accounting for a substantial proportion of cancer-related morbidity and mortality. Its prognosis is strongly dependent on the stage at diagnosis, with early-stage disease associated with improved survival and the possibility of minimally invasive treatment. Evidence from populations in which endoscopic screening has been implemented suggests that screening may contribute to reducing gastric cancer-related mortality, although its applicability varies according to epidemiological context [3].

*H. pylori* infection is a major risk factor for non-cardia gastric adenocarcinoma, particularly the intestinal histological subtype. The progression described in the Correa cascade, in which chronic gastric inflammation may progress through atrophic gastritis, intestinal metaplasia, and dysplasia to carcinoma, highlights the importance of identifying premalignant gastric conditions [4]. Most individuals with *H. pylori* infection remain asymptomatic. In this context, the identification and eradication of *H. pylori* are important components of gastric cancer prevention strategies, although the benefits and cost-effectiveness of population-based approaches depend on the underlying risk and healthcare setting [4].

Gastric cancer exhibits considerable geographic variation in incidence across countries and regions. In Brazil, the National Cancer Institute (INCA) estimated 21,480 new cases annually for the 2023-2025 period. In Minas Gerais and Belo Horizonte, the estimated crude incidence rates for 2023 were 10.33 and 11.43 cases per 100,000 inhabitants, respectively [5]. Although population-based endoscopic screening is more established in certain high-incidence countries, its implementation in other epidemiological settings remains controversial and depends on factors such as population risk, cost-effectiveness, and healthcare resource availability.

Emerging evidence has explored the potential role of EGD in gastric cancer screening and prevention across different epidemiological settings [6,7]. Opportunistic EGD performed during elective colonoscopy has been proposed as a strategy to identify upper gastrointestinal abnormalities without requiring a separate endoscopic session. Previous studies have suggested that this combined approach may offer logistical advantages and could be cost-effective in selected populations [1,4,8]. However, its clinical value depends on several factors, including the prevalence and relevance of detected lesions, procedural risks, healthcare costs, and subsequent patient outcomes. Furthermore, evidence remains limited, and findings from selected populations may not be directly generalizable to other healthcare settings [8].

Therefore, this study aimed to evaluate the diagnostic yield of opportunistic EGD performed during elective colonoscopy in asymptomatic patients at a tertiary endoscopy center in Brazil, focusing on the frequency and characteristics of clinically significant upper gastrointestinal findings.

## Materials and methods

Study design and setting

This was a retrospective cross-sectional study based on the analysis of electronic medical records from the “MV PEP SOUL” and “Philips Radiology Information” systems. The study included asymptomatic patients who underwent opportunistic EGD during elective colonoscopy at a tertiary endoscopy center (Hospital Mater Dei Santo Agostinho, Belo Horizonte, Brazil).

The diagnostic yield of opportunistic EGD was defined as the proportion of patients with at least one clinically significant finding. Potential clinical impact was considered in terms of whether the identified conditions could warrant medical treatment, further diagnostic evaluation, outpatient follow-up, endoscopic surveillance, or therapeutic intervention. As systematic postprocedural follow-up was not performed, actual changes in patient management were not assessed.

Clinically significant findings were defined as malignant neoplasms, premalignant lesions (Barrett’s esophagus, gastric intestinal metaplasia, gastric atrophy, and low- or high-grade dysplasia), peptic ulcers, subepithelial lesions, *H. pylori*-associated gastritis, erosive esophagitis classified as Los Angeles grades B, C, or D, infectious esophagitis, hiatal hernia, and selected additional lesions, including gastrointestinal adenomas and esophageal papillomas.

Endoscopic findings and corresponding histopathological results were reviewed. During endoscopic procedures, systematic biopsies of the gastric antrum and body were routinely performed. In cases of suspected gastric atrophy or intestinal metaplasia, at least two biopsy specimens were obtained from the antrum/incisura, with the incisura biopsied separately in some cases, and two additional specimens were obtained from the gastric body. Targeted biopsies were also performed according to esophageal, gastric, or duodenal findings. *H. pylori* infection, gastric atrophy, intestinal metaplasia, and dysplasia were diagnosed by histopathological examination of the biopsy specimens. Histological analysis was conducted by experienced gastrointestinal pathologists at our institution.

Participants

Asymptomatic patients who underwent opportunistic EGD during elective colonoscopy between March 2023 and March 2025 were eligible for inclusion. Patients were retrospectively identified through institutional electronic systems, and all procedures performed during the study period were screened for eligibility. Eligible patients were consecutively included according to the predefined inclusion and exclusion criteria.

All patients had undergone a clinical consultation before scheduling the procedures, with medical history and gastrointestinal symptoms documented in the electronic medical records, as well as a pre-anesthetic evaluation. Asymptomatic status was retrospectively verified by reviewing both records. Gastrointestinal symptoms and other clinical indications for diagnostic EGD included gastrointestinal bleeding, abdominal pain, reflux symptoms, dysphagia, food impaction, dyspepsia, diarrhea, weight loss, and anemia.

Medical records with incomplete information for the analyzed variables were excluded. No repeated procedures were identified among the included patients. The study inclusion and exclusion criteria are presented in Table 1.

**Table 1:** Inclusion and exclusion criteria

Inclusion criteria	Exclusion criteria
All asymptomatic patients undergoing opportunistic EGD during elective colonoscopy within the study period were eligible.	Gastrointestinal symptoms
	Known gastrointestinal diseases
	Family history of gastrointestinal malignancy
	Prior upper gastrointestinal surgery or procedures performed for endoscopic surveillance
	Current use of proton pump inhibitors
	Incomplete medical records

Sample size calculation

The sample size was calculated to estimate a proportion in a descriptive study. Assuming an expected proportion of 0.50, a 95% confidence level, and a 5% margin of error, a minimum sample size of 384 patients was required.

Statistical analysis

Qualitative variables were described using absolute and relative frequencies, while quantitative variables were summarized using minimum and maximum values, mean, standard deviation (SD), median, and interquartile range.

The diagnostic yield was calculated as the proportion of patients with at least one clinically significant finding among all included participants, with the corresponding 95% confidence interval (CI). As individual patients could present multiple findings, diagnostic categories were not considered mutually exclusive.

No imputation of missing data was performed, as medical records with incomplete information for the analyzed variables were excluded from the study.

All analyses were performed using R version 4.5.0 (R Core Team, Vienna, Austria) through RStudio version 2025.05.0 (Posit Software, PBC, Boston, MA, USA).

Ethics approval

This study was approved by the Research Ethics Committee of Hospital Mater Dei S.A. (CAAE: 88452625.8.0000.5128). Due to the retrospective nature of the study, in which data were obtained through medical record review, the requirement for informed consent was waived by the Institutional Review Board.

Availability of data and materials

The data supporting the findings of this study were obtained from the institutional electronic medical record systems (“MV PEP SOUL” and “Philips Radiology Information”) of Hospital Mater Dei Santo Agostinho. The study dataset is not publicly available due to patient confidentiality and institutional restrictions. Data may be made available by the corresponding author upon reasonable request, subject to institutional authorization and applicable ethical and privacy requirements.

## Results

Study population

A total of 421 (100%) asymptomatic patients who underwent opportunistic EGD during elective colonoscopy were included. Of these, 269 (63.9%) were female, and the mean age was 58.4 ± 10.4 years. These characteristics are summarized in Table 2. At least one clinically significant finding was identified in 188 patients' (44.7%) examinations (95% CI: 39.8%-49.5%).

**Table 2:** Sample characteristics

Variables	Statistics
Sex	
Female	269 ​​​​(63.9%)
Male	152 (36.1%)
Age at examination (years)	
Minimum/maximum	29.0/83.0
Median (Q1; Q3)	58.0 (51.0; 66.0)
Mean (SD)	58.4 (10.4)
Age group	
29–44 years	36 (8.6%)
45–59 years	198 (47.0%)
≥60 years	187 (44.4%)

Gastric findings

Gastritis was the most frequent finding, identified in 293 patients (69.6%). Among patients with gastritis, 102 (34.8%) were positive for *H. pylori*, corresponding to 24.2% of the overall study population. Premalignant gastric lesions were detected in 74 (17.6%) patients, including intestinal metaplasia in 45 (10.7%), gastric atrophy in 27 (6.4%), and low-grade dysplasia in 2 (0.5%). Gastric polyps were identified in 140 (33.3%) patients, most of which were fundic gland polyps. Subepithelial lesions were identified in 14 (3.3%) patients.

Notably, gastric carcinoma and mucosa-associated lymphoid tissue (MALT) lymphoma were each diagnosed in 1 (0.2%) patient. Peptic ulcer disease was identified in 6 (1.4%) patients, of whom 3 (50.0%) were positive for *H. pylori*. These endoscopic findings are detailed in Table 3.

**Table 3:** Distribution of gastric mucosal findings MALT: mucosa-associated lymphoid tissue

Gastric findings	Statistics
Gastritis	293 (69.6%; 95% CI 65.2–74.0)
Reactive gastropathy	1 (0.24%)
Chronic gastritis	292 (69.4%)
*H. pylori*-related gastritis	102 (24.2%)
Ulcerative lesions	6 (1.4%; 95% CI 0.3–2.5)
Atrophy	27 (6.4%; 95% CI 4.1–8.7)
Intestinal metaplasia	45 (10.7%; 95% CI 7.7–13.7)
Low-grade dysplasia	2 (0.47%; 95% CI 0.0–1.5)
Gastric polyps	140 (33.3%; 95% CI 28.8–37.8)
Fundic gland polyps	92 (21.9%)
Hyperplastic polyps	26 (6.2%)
Inflammatory polyps	18 (4.3%)
Adenoma	1 (0.24%)
Polypoid fold	3 (0.71%)
Subepithelial lesions	14 (3.3%; 95% CI 1.6–5.0)
Gastric carcinoma (Lauren mixed type)	1 (0.2%)
MALT lymphoma	1 (0.2%)

Duodenal findings

Duodenal abnormalities were observed in a subset of patients, with bulboduodenitis identified in 26 (6.2%) patients. Other findings included duodenal polyps, subepithelial lesions, and duodenal ulcer; the respective frequencies are presented in Table 4. Tubular adenoma with low-grade dysplasia in the second portion of the duodenum was identified in 2 (0.5%) patients, and adenoma of the minor duodenal papilla was detected in 1 (0.2%) patient. These characteristics are demonstrated in Table 4.

**Table 4:** Distribution of duodenal mucosal findings

Duodenal findings	Statistics
Bulboduodenitis	26 (6.2%; 95% CI 3.9–8.5)
		Bulbar ulcer	1 (0.2%; 95% CI 0.0–1.5)
Bulbar scarring deformity	10 (2.4%; 95% CI 0.9–3.9)
		Duodenal polyps	12 (2.9%; 95% CI 1.5–4.9)
Gastric mucosal heterotopia	4 (1.0%)
Adenoma	2 (0.5%)
Hyperplastic polyp	2 (0.5%)
Inflammatory polyp	2 (0.5%)
Brunner gland hyperplasia	1 (0.2%)
Polypoid lesion	1 (0.2%)
Minor papilla adenoma	1 (0.2%; 95% CI 0.0–1.5)
		Subepithelial lesions	5 (1.2%; 95% CI 0.2–2.2)

Esophageal findings

Esophagitis was identified in 134 (31.8%) patients, with severity classified according to the Los Angeles classification (Table 5). Hiatal hernia was identified in 29 (6.9%) patients, and Barrett’s esophagus was confirmed in 3 (0.7%) patients. Other findings included esophageal papillomas, inflammatory polyps, subepithelial lesions, and esophageal candidiasis. Endoscopic findings are detailed in Table 5.

**Table 5:** Distribution of esophageal mucosal findings

Esophageal findings	Statistics
Esophagitis	134 (31.8%; 95% CI 27.4–36.2)
Grade A	99 (23.5%)
Grade B	29 (6.9%)
Grade C	5 (1.2%)
Grade D	1 (0.24%)
Hiatal hernia	29 (6.9%; 95% CI 4.5–9.3)
Barrett’s esophagus	3 (0.7%; 95% CI 0.0–1.5)
Polypoid lesions	10 (2.4%; 95% CI 0.9–3.9)
Papillomas	7 (1.7%)
Inflammatory polyps	3 (0.7%)
Subepithelial lesion	1 (0.2%; 95% CI 0.0–1.5)
Esophageal candidiasis	3 (0.7%; 95% CI 0.0–1.5)

Findings according to *H. pylori* status

Among the 293 patients with gastritis, 102 (34.8%) were positive for *H. pylori*. *H. pylori* infection was also associated with peptic ulcer disease and inflammatory duodenal findings. The distribution of endoscopic findings is shown in Table 6.

**Table 6:** Gastrointestinal findings according to H. pylori status Percentages calculated by rows.

Findings	*H. pylori* negative	*H. pylori* positive
Gastritis	191 (65.2%)	102 (34.8%)
Ulcerative lesions	3 (50%)	3 (50%)
Atrophy	18 (66.7%)	9 (33.3%)
Metaplasia	34 (75.6%)	11 (24.4%)
Hyperplastic polyps	24 (92.3%)	2 (7.7%)
Inflammatory polyps	9 (50%)	9 (50%)

## Discussion

In this study, opportunistic EGD performed during elective colonoscopy in asymptomatic patients demonstrated a substantial diagnostic yield, with at least one clinically significant finding identified in 188 patients (44.7%). These findings included premalignant and malignant lesions, *H. pylori* infection, and other upper gastrointestinal abnormalities that could potentially warrant treatment, further diagnostic evaluation, or endoscopic surveillance. However, as systematic postprocedural follow-up was not performed, actual changes in clinical management and subsequent patient outcomes could not be assessed. Therefore, the findings should be interpreted as evidence of diagnostic yield rather than demonstrated clinical benefit.

Our findings suggest that a considerable burden of upper gastrointestinal abnormalities may remain undetected in asymptomatic individuals. Reliance exclusively on symptom-based indications may therefore fail to identify some clinically relevant conditions, including premalignant and malignant lesions. Nevertheless, the detection of additional abnormalities does not necessarily establish the benefit of routine screening, which also depends on the clinical significance of the findings and the balance between potential benefits, risks, and costs.

An important finding of this study was the detection of premalignant gastric lesions in 74 patients (17.6%), including intestinal metaplasia, gastric atrophy, and dysplasia. Global estimates of the prevalence of gastric premalignant conditions are highly variable and remain insufficiently characterized, and our findings are consistent with previously reported prevalence rates in populations from regions with low to intermediate gastric cancer risk [9]. Early identification of these lesions may allow risk stratification and consideration of endoscopic surveillance according to individual risk and current recommendations. Notably, recent meta-analyses have demonstrated that the progression rates of precursor lesions in low-incidence countries are comparable to those observed in medium- and high-incidence regions, suggesting that the clinical relevance of these lesions extends beyond traditionally high-risk populations [10]. However, whether their detection through opportunistic EGD improves long-term clinical outcomes requires further investigation.

The prevalence of *H. pylori* infection, identified in 102 patients (24.2%), is consistent with data from Western populations [11,12]. Given its central role in gastric carcinogenesis, the detection of *H. pylori* in asymptomatic individuals may provide an opportunity for eradication therapy, which has been associated with a reduced risk of gastric cancer [13]. Nevertheless, treatment initiation, successful eradication, and subsequent clinical outcomes were not evaluated in the present study.

Although uncommon, the identification of one case of gastric carcinoma (0.2%) and one case of MALT lymphoma (0.2%) illustrates the ability of opportunistic EGD to detect clinically silent malignancies. However, the small number of malignant lesions and the absence of longitudinal follow-up prevent conclusions regarding the effect of this strategy on cancer-related morbidity or mortality.

In addition to gastric findings, relevant esophageal and duodenal abnormalities were identified. A total of 20 subepithelial lesions (4.8%) were detected, some of which may warrant further evaluation with endoscopic ultrasound or endoscopic surveillance, depending on their characteristics and individual risk assessment [14]. Gastrointestinal polyps, including adenomas, and peptic ulcer disease were also identified. These findings may have implications for subsequent clinical management, although the proportion of patients who actually underwent additional investigations, treatment, or surveillance was not assessed.

Regarding esophageal conditions, 35 patients (8.3%) presented with Los Angeles grade B, C, or D esophagitis, findings that may support the diagnosis of gastroesophageal reflux disease (GERD) in the appropriate clinical context. Hiatal hernias were also detected and may be associated with increased reflux burden [15]. Barrett’s esophagus was identified in a small proportion of patients and may warrant endoscopic surveillance according to established risk-based recommendations. In addition, esophageal candidiasis was detected, a finding that may indicate the need for antifungal treatment and assessment of underlying predisposing factors. However, subsequent management was not evaluated in this study.

From a practical perspective, performing opportunistic EGD during an already scheduled colonoscopy may offer logistical advantages by avoiding a separate endoscopic session and potentially reducing the need for additional sedation and healthcare visits. Previous cost-utility analyses have suggested that upper gastrointestinal endoscopic screening may be cost-effective when combined with colonoscopy screening in individuals aged 50 to 75 years [4], including in European countries with intermediate gastric cancer risk and in low-incidence settings such as the United States [1]. Recent evidence from an intermediate-risk Western population has also suggested that opportunistic EGD performed during colorectal cancer screening is feasible, acceptable to patients, and associated with a substantial diagnostic yield [16]. In addition to premalignant lesions, previous studies have reported the detection of gastroduodenal ulcers, Barrett’s esophagus, and esophageal varices using this combined approach [1,15]. Nevertheless, these potential advantages cannot be directly extrapolated to the present study, as procedural costs, cost-effectiveness, patient acceptance, adherence, and comparative resource utilization were not evaluated.

Several limitations should be acknowledged. First, the retrospective, single-center design introduces the possibility of selection bias and limits the generalizability of the findings. Although asymptomatic status was verified through available clinical and pre-anesthetic records, retrospective symptom assessment may be subject to incomplete documentation and misclassification. The exclusion of patients with incomplete medical records may also have introduced selection bias. Furthermore, the absence of a control group undergoing colonoscopy alone prevents direct assessment of the incremental clinical value of opportunistic EGD compared with usual care. Routine gastric biopsies may have increased the detection of histological abnormalities, potentially contributing to detection bias and limiting comparability with studies using different biopsy protocols. Additionally, not all detected abnormalities necessarily require treatment or surveillance, raising the possibility of overdiagnosis and unnecessary subsequent investigations.

Another important limitation is the absence of systematic postprocedural follow-up. Consequently, actual changes in medical treatment, additional diagnostic procedures, endoscopic surveillance, and long-term patient outcomes could not be quantified. Potential procedure-related harms, additional costs arising from incidental findings, patient acceptance, and adherence to recommended follow-up were also not evaluated. Moreover, detailed information on potential confounding factors, including smoking, alcohol consumption, and obesity, was unavailable. Finally, although regional gastric cancer incidence estimates provide relevant epidemiological context, crude incidence rates alone do not establish a formal intermediate-risk classification based on age-standardized incidence.

Despite these limitations, the study provides descriptive data on upper gastrointestinal findings in a consecutively selected population of asymptomatic patients undergoing opportunistic EGD during elective colonoscopy. The use of systematic gastric biopsies and histopathological evaluation allowed the identification of conditions that might otherwise have remained undetected.

Overall, opportunistic EGD performed during elective colonoscopy demonstrated a substantial diagnostic yield in this population, including the detection of premalignant and malignant upper gastrointestinal lesions. However, these findings do not establish that routine opportunistic EGD improves clinical outcomes or is cost-effective. Prospective comparative studies incorporating longitudinal follow-up, patient-centered outcomes, procedural safety, and economic evaluation are needed to determine which populations may benefit most from this approach.

## Conclusions

Opportunistic EGD performed during elective colonoscopy in asymptomatic patients demonstrated a substantial diagnostic yield, identifying clinically significant findings, including *H. pylori* infection, premalignant gastric lesions, and, less frequently, malignant neoplasms. These findings may have implications for subsequent clinical management, including medical treatment, further diagnostic evaluation, and endoscopic surveillance.

The results suggest that clinically relevant upper gastrointestinal abnormalities may remain undetected in asymptomatic individuals. However, the absence of systematic follow-up and a comparison group prevents conclusions regarding actual changes in patient management or improvements in clinical outcomes. Further prospective comparative studies incorporating longitudinal follow-up, procedural safety, and cost-effectiveness analyses are needed to determine the clinical value and appropriate role of opportunistic EGD during elective colonoscopy.
