# Correction: Diagnostic Yield of Opportunistic Upper Gastrointestinal Endoscopy During Elective Colonoscopy in Asymptomatic Patients: A Retrospective Cross-Sectional Study

**DOI:** 10.7759/cureus.c495

**Published:** 2026-10-21

**Authors:** Caroline Assis Aleixo Chaves Freitas, Sandro Rodrigues Chaves, Rafael P Pessoa, Lucas Gallo de Alvarenga Mafra, Júlia Gallo de Alvarenga Mafra, Eduardo Seubert Coelho Vieira, Gabriela Vieira Brito, Giovanna De Giuli, Rodrigo Roda

**Affiliations:** 1 Endoscopy Division, Rede Mater Dei de Saúde, Belo Horizonte, BRA; 2 Gastroenterology Division, Rede Mater Dei de Saúde, Belo Horizonte, BRA; 3 Endoscopy Division, Faculdade Ciências Médicas de Minas Gerais, Belo Horizonte, BRA; 4 Endoscopy Division, Instituto Alfa de Gastroenterologia, Hospital das Clínicas da Universidade Federal de Minas Gerais, Belo Horizonte, BRA

This article has been corrected to replace the previously published affiliations for Dr. Rodrigo Roda and Dr. Sandro Rodrigues Chaves.

